# Brain volumes discriminate clinical dementia rating scale categories

**DOI:** 10.1038/s41598-025-23418-9

**Published:** 2025-11-13

**Authors:** Peka Christova, Lisa M. James, Apostolos P. Georgopoulos

**Affiliations:** 1 The Neuroimaging Research Group, Brain Sciences Center, Department of Veterans Affairs Health Care System, Minneapolis, MN 55417 USA; 2 Department of Neuroscience, University of Minnesota Medical School, Minneapolis, MN 55455 USA; 3 Department of Psychiatry, University of Minnesota Medical School, Minneapolis, MN 55455 USA; 4 Institute for Health Informatics, University of Minnesota Medical School, Minneapolis, MN 55455 USA

**Keywords:** Dementia, Brain atrophy, OASIS project, Clinical dementia rating (CDR), Brain volume, MRI, Neuroscience, Anatomy, Diseases

## Abstract

Brain atrophy is well documented in various kinds of dementia, particularly in Alzheimer’s disease. Here, we evaluated gray matter volume of 87 cortical and subcortical areas in 460 individuals characterized according to the Clinical Dementia Rating (CDR) as cognitively unimpaired (*n* = 352), undetermined (*n* = 72), or mild dementia (*n* = 36). We found a highly significant correspondence between increased dementia severity and reduced brain volume, particularly for the amygdala and temporal cortical areas, including the hippocampus, middle temporal gyrus, and inferior temporal gyrus. The negative correlation between brain volumes and dementia severity was significantly stronger in men than women, and in apolipoprotein E4 carriers than non-carriers. Brain volumes discriminated between cognitively unimpaired and mild dementia cases with high accuracy; application of those classification functions to the undetermined group resulted in two distinct groups, one resembling the cognitively unimpaired Control group and another resembling the Dementia group. These findings highlight the correspondence between clinical dementia stages and objective brain volume measures, and point to the potential clinical utility of adjunctive structural brain measures to identify individuals with memory complaints who may be at risk of dementia.

## Introduction

Dementia is a clinical syndrome characterized by progressive decline in cognitive functions accompanied by impairments in activities of daily living and alterations in behavior and personality. Alzheimer’s dementia (AD) is the most common type accounting for 60–70% of dementia cases^1^; nearly 11% of adults age 65 and over in the United States has AD^2^. AD progresses along a continuum from preclinical to prodromal “mild cognitive impairment” and across dementia stages (mild, moderate and severe)^3^. Recently, an emphasis has been placed on the earliest stages of the continuum as the optimal window for preventive action before irreparable brain changes develop^4^. In the pre-clinical stage, individuals may show evidence of AD pathology (e.g., amyloid beta) in the absence of symptoms^5^. For those who progress to the next stage of prodromal dementia (also referred to as mild cognitive impairment), subtle problems with memory or language may develop although not to the extent that they interfere with daily functioning; about one-third of individuals with mild cognitive impairment develop dementia within 5 years^6^.

 Extensive evidence has documented brain atrophy associated with dementia^7^ that is distinct from atrophy due to normal aging^8,9^. Along the AD continuum, gross structural changes in the brain are commensurate with symptom progression^10–12^. Indeed, cerebral atrophy is more closely related to cognitive impairment in AD than fluid biomarkers that may be present decades before symptom onset^13^, highlighting the relevance of progressive cerebral atrophy to dementia stages. Extensive research, reviewed elsewhere^7,12^, has demonstrated a predictable pattern of AD-associated cortical atrophy starting in the medial temporal lobe (e.g., hippocampus, entorhinal cortex) and expanding to parietal and then other cortical regions. Subcortical atrophy has similarly been documented in dementia albeit with fewer studies and somewhat inconsistent findings for certain structures^7,12,14–17^. Documented patterns of atrophy correspond with the topographic distribution and progression of neurofibrillary tangles, neuronal counts, and Braak stage^18–21^. Furthermore, global and specific (e.g. hippocampal) cortical and subcortical brain atrophy has been associated with cognitive decline and impairment^22–24^.

In research and clinical settings, cognitive impairment is often based on assessments that evaluate dementia-characteristic decrements in cognitive functions and activities of daily living. One widely used tool is the Clinical Dementia Rating (CDR) Scale^25^ which utilizes patient and informant interviews to rate decrements in performance from premorbid levels in several cognitive (memory, orientation, judgment and problem-solving) and functional (community affairs, home and hobbies, and personal care) domains from which a global score is derived. A CDR score of 0 indicates no dementia, 0.5 indicates questionable dementia, 1 is mild dementia, 2 is moderate, and 3 indicates severe dementia. The CDR is a reliable and validated measure^26,27^ with good (> 90%) sensitivity and specificity in distinguishing older adults with or without mild cognitive impairment (MCI) and dementia^28^. Research has shown that CDR staging corresponds with neuropsychiatric symptoms and decrements in Activities of Daily Living (ADLs)^29^ and brain age scores^30^, white matter integrity^31^, and brain atrophy^14,32^. Here, we evaluated cortical and subcortical gray matter volume in individuals characterized as cognitively unimpaired (CDR 0), in the earliest stages of dementia (i.e., questionable or undetermined dementia (CDR 0.5), and with mild dementia (CDR 1), and with regard to sex and apolipoprotein E (apoE). Since age-related brain atrophy has been observed among healthy adults^9^, we controlled for age in the present study.

The main aims of this study were to (a) quantify the relations between the volumes of 87 brain areas (70 cortical, 17 subcortical) and the CDR score (controlling for sex, age, education and race), (b) assess the effect of apoE genetic makeup on these relations, and (c) test the hypothesis that the undetermined group (CDR 0.5) is made up of two distinct subgroups (akin to control and mild dementia main groups) that could be distinguished by the set of brain volumes analyzed.

## Materials and methods

### Participants

We analyzed data provided by OASIS-3 (Open Access Series of Imaging Studies): Longitudinal Multimodal Neuroimaging, www.oasis-brains.org. (https://sites.wustl.edu/oasisbrains/). The OASIS-3 is a large and publicly available set of imaging and cognitive data^33^. Imaging data are in NIFTI format.

Participant dementia severity was evaluated by semi-structured interview that generated the Clinical Dementia Rating (CDR) score in accordance with established scoring^25^. The questionnaire includes the following subfields: Memory, Orientation, Judgment & problem solving, Community affairs, Home & hobbies, Personal care. The total and sum of the scores of participants are available. A CDR total score of 0 is considered a healthy control participant with normal cognition, a CDR score of 0.5 is considered undetermined, a CDR score of 1 corresponds to mild dementia. Education was coded as number of years of education. Race was also coded. ApoE genotype was available for 457/460 participants.

In this study we used only data acquired with the same Siemens Trio TIM (Total Imaging Matrix) 3 T scanner, to eliminate possible differences due to different magnets. Acquired structural images were T1-weighted (T1w), T2-weighted (T2w), and gradient field map (FM, magnitude and phase difference). There were 460 participants fulfilling those requirements (207 men and 253 women). They comprised 352 control (CDR 0; 140 men, 212 women), 72 undetermined (CDR 0.5; 42 men, 30 women), and 36 mild AD (CDR 1; 25 men, 11 women).

### Structural MRI data

We used the open-source Human Connectome Project pipeline 4.7.0 processing software tool (https://github.com/Washington-University/HCPpipelines) in 3 consecutive steps (PreFreeSurfer, FreeSurfer and PostFreeSurfer structural pipelines)^34^ using FreeSurfer (v 6.0, https://surfer.nmr.mgh.harvard.edu). Our purpose in using the HCP pipelines is that they provide high-quality volume data using both T1w and T2w images. The field map was used to remove readout distortion in the T1w and T2w images, which improved their alignment^34^. As stated in the FreeSurfer website *“it is essential to process all participants with the same version of FreeSurfer*,* on the same OS platform and vendor*,* and for safety*,* even the same version of the OS*” which is what we did. This was the reason to include only cross-sectional participant data acquired with the same Siemens scanner, processed data with the same version of FreeSurfer, and on the same computer cluster with the same OS.

After successful processing of raw data, we extracted the volumes of total gray matter, total cortical gray matter, left cortical and right cortical gray matter. Additionally, volumes of the following 70 areas of the Desikan-Killiany atlas (35 areas in the left and 35 areas in the right hemisphere) were extracted^35^: banks of the superior temporal sulcus, caudal anterior cingulate gyrus, caudal middle frontal gyrus, cuneus, entorhinal gyrus, frontal pole, fusiform gyrus, hippocampus, inferior frontal gyrus pars opercularis, inferior frontal gyrus pars orbitalis, inferior frontal gyrus pars triangularis, inferior parietal lobule, inferior temporal gyrus, insula, isthmus of cingulate gyrus, lateral occipital gyrus, lateral orbitofrontal gyrus, lingual gyrus, medial orbitofrontal gyrus, middle temporal gyrus, paracentral gyrus, parahippocampal gyrus, pericalcarine gyrus, postcentral gyrus, posterior cingulate gyrus, precentral gyrus, precuneus, rostral anterior cingulate gyrus, rostral middle frontal gyrus, superior frontal gyrus, superior parietal lobule, superior temporal gyrus, supramarginal gyrus, temporal pole, transverse temporal gyrus. The volumes of following subcortical nuclei were extracted: accumbens, amygdala, brainstem, caudate, cerebellum cortex, pallidum, putamen, thalamus, and ventral diencephalon^36^.

The extracted volume (*V*) of each area was standardized as a percent of Estimated Total Intracranial Volume (eTIV):

1 $$\:\text{N}\text{o}\text{r}\text{m}\text{a}\text{l}\text{i}\text{z}\text{e}\text{d}\:\text{v}\text{o}\text{l}\text{u}\text{m}\text{e}\:\left(\text{\%}\right):\:{V}^{{\prime\:}}=100\:\frac{V}{eTIV}$$

$\:V^{\prime\:}$ re-expresses the raw volume (originally in mm^3^) as a fraction (%) of the total intracranial (skull) volume to standardize it across participants with different skull sizes.

### Data analyses

Standard statistical methods were used to analyze the data, including descriptive statistics, t-test, analysis of covariance (ANCOVA), repeated measures analysis of variance, partial correlation, and linear discriminant analysis (LDA) with equal priors. The IBM-SPSS statistical package (version 30) was used for statistical analyses. All reported P values are two-sided.

## Results

### Descriptive statistics

Descriptive statistics for the various measures used are given in Table 1.

**Table 1:** Descriptive statistics for the measures used.

		CDR group		
		0	0.5	1
N		352	72	36
Age (y)	Mean (SEM)	68.49 (0.48)	75.92 (0.81)	77.43 (1.44)
Sex	Men (M), Women (W)	140 M, 212 W	42 M, 30 W	25 M, 11 W
Education (y)	Years (SEM)	16.9 (0.138)	14.72 (0.320)	14.42 (0.464)
Race	White (W), Black (B), Asian (A)	296 W, 53 B, 3 A	59 W, 13 B	26 W, 10 B
MMSE		29.18 (0.06)	26.31 (0.347)	22.78 (0.703)
Informant sex		121 M, 228 W	17 M, 51 W	5 M, 30 W
Informant Relation	1-Spouse/partner | 2-Child | 3-Sibling | 4-Other relative | 5-Friend/neighbor | 6-Paid caregiver/provider | 7-Other	1 (185), 2 (75) 3 (20), 4 (11) 5 (47), 7 (4)	1 (44), 2 (17) 3 (1), 4 (2) 5 (3), 7 (1)	1 (27), 2 (4) 3(3), 5 (1)
ApoE	ε2/ε2	3	0	0
	ε2/ε3	43	8	3
	ε2/ε4	13	4	0
	ε3/ε3	176	30	12
	ε3/ε4	102	24	17
	ε4/ε4	14	5	3

### Group comparisons

#### Age

**Fig. 1:**
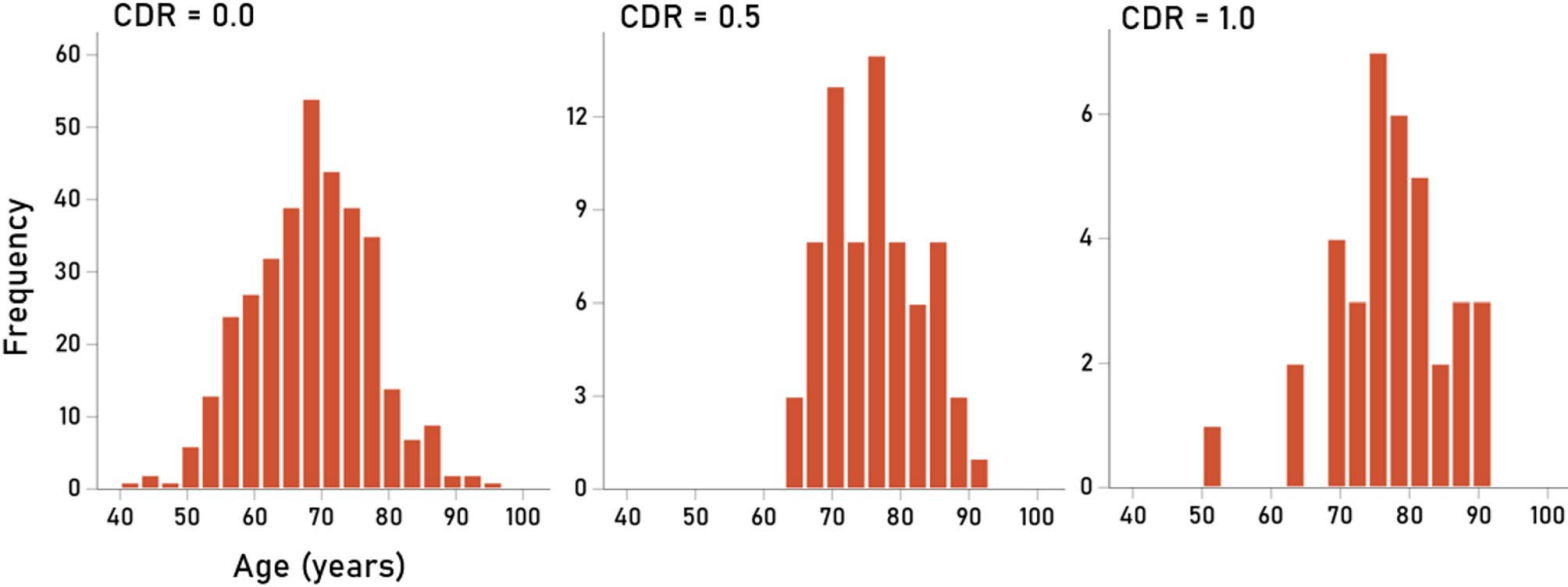
Frequency distributions of age for the 3 CDR groups, as indicated. N(CDR 0) = 352, N(CDR 0.5) = 72, N(CDR 1) = 36.

The overall age of study participants was 70.35 ± 0.43 y (mean ± SEM; *N* = 460). Figure 1 shows the frequency distribution of age in the three CDR groups (Table 1). Pairwise age differences among the three CDR groups were assessed using a univariate ANOVA where age was the dependent variable, and Group (CDR) and Sex were independent variables. We found the following. (a) The Group (CDR) main effect was statistically significant (F_[2,454]_ = 30.02, *P* < 0.001); mean age was significantly smaller in the Control group than the CDR 0.5 or CDR 1 groups (*P* < 0.001, Bonferroni corrected for multiple comparisons, whereas mean ages did not differ significantly between CDR 0.5 and CDR 1 groups (*P* = 1.0, Bonferroni adjusted). (b) The Sex main effect was not significant (F_[1,454]_ = 1.46, *P* = 0.228). And (c) the Group x Sex interaction was not statistically significant (F_[2,454]_ = 1.32, *P* = 0.267).

### Education

Descriptive statistics for Education are given in Table 1. The Control group (CDR 0) had ~ 2 years more education, whereas the years of education for CDR 0.5 and CDR 1 were very similar. We assessed the CDR-education relation by performing a univariate ANOVA, where Education (years) was the dependent variable and CDR was a fixed group factor. We found the following. (a) The CDR main effect was statistically significant (F_[2,460]_ = 13.12, *P* < 0.001). (b) Education in the CDR 0 group was significantly higher than that in the CDR 0.5 and CDR 1 groups (*P* < 0.001 for both comparisons, Bonferroni adjusted). (c) Education did not differ significantly between CDR 0.5 and CDR 1 groups (*P* = 1.0, Bonferroni adjusted). (d) The results of the Education CDR group comparisons above remained the same when Sex and Race were added as fixed factors and Age as a covariate in the ANOVA.

### Race

The Race distribution among the three CDR groups is shown in Table 1. This did not differ significantly among the CDR groups (Pearson Chi-Square test, χ_[4]_ = 4.8, *P* = 0.308).

### ApoE

The distribution of apoE genotype among the three CDR groups is shown in Table 1. This did not differ significantly among the CDR groups (Pearson Chi-Square test, χ_[10]_ = 11.79, *P* = 0.299).

### MMSE

Descriptive statistics for MMSE are given in Table 1. Mean MMSE decreased with increasing CDR: MMSE (CDR 0) > MMSE (CDR 0.5) > MMSE (CDR 1). All pairwise comparisons were statistically significant in a univariate ANOVA (*P* < 0.001 for all, Bonferroni corrected). The negative partial correlation $\:{r}_{p}$ between MMSE and CDR (controlling for Sex, Age, Education, and Race) was also statistically significant ($\:{r}_{p}$ = −0.641, *P* < 0.001, *N* = 460).

### Cortex

#### Normalized cortical volumes $\:V^{\prime\:}$

The effect of various factors on cortical gray $\:V^{\prime\:}$ was assessed by performing a univariate ANCOVA where $\:V^{\prime\:}$ was the dependent variable, CDR and Race were fixed (categorical) factors, and Sex, Age and Education were covariates. The results are shown in Table 2. It can be seen that only CDR, Sex and Age had statistically significant effects.

**Table 2:** Effects of various factors on normalized cortical Gray volume $\:V^{\prime\:}$ as assessed by the ANCOVA described in text. DF, degrees of freedom; F, value of F statistic; P, probability value.

Factor	DF	F	P
CDR	2	22.442	< 0.001
Sex	1	35.35	< 0.001
Age	1	300.782	< 0.001
Education	1	0.165	0.685
Race	2	1.094	0.336
CDR* Race	2	0.195	0.828
Error	444		
Total	459		

### CDR and normalized cortical volumes

**Fig. 2:**
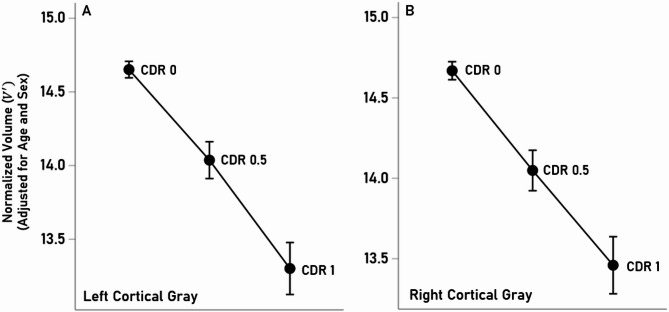
Mean (± SEM) normalized ($\:V^{\prime\:}$) gray matter volumes for the left (**A**) and right (**B**) hemispheres are plotted against the CDR score.

Normalized cortical gray matter volumes ($\:V^{\prime\:}$) decreased systematically with increasing CDR score: $\:V^{\prime\:}$(CDR 1) < $\:V^{\prime\:}$(CDR 0.5) < $\:V^{\prime\:}$(CDR 0). More specifically, we found highly significant negative partial correlations ($\:{r}_{p}$) between cortical volumes and CDR scale (controlling for sex and age), as follows. Left cortical gray (Fig. 2A), $\:{r}_{p}$ = −0.342 (*P* < 0.001, *N* = 460); (b) right cortical gray (Fig. 2B), $\:{r}_{p}$ = −0.317 (*P* < 0.001, *N* = 460). These correlations did not differ significantly (z = 0.424, *P* = 0.672).

### CDR and cortical areas

**Fig. 3:**
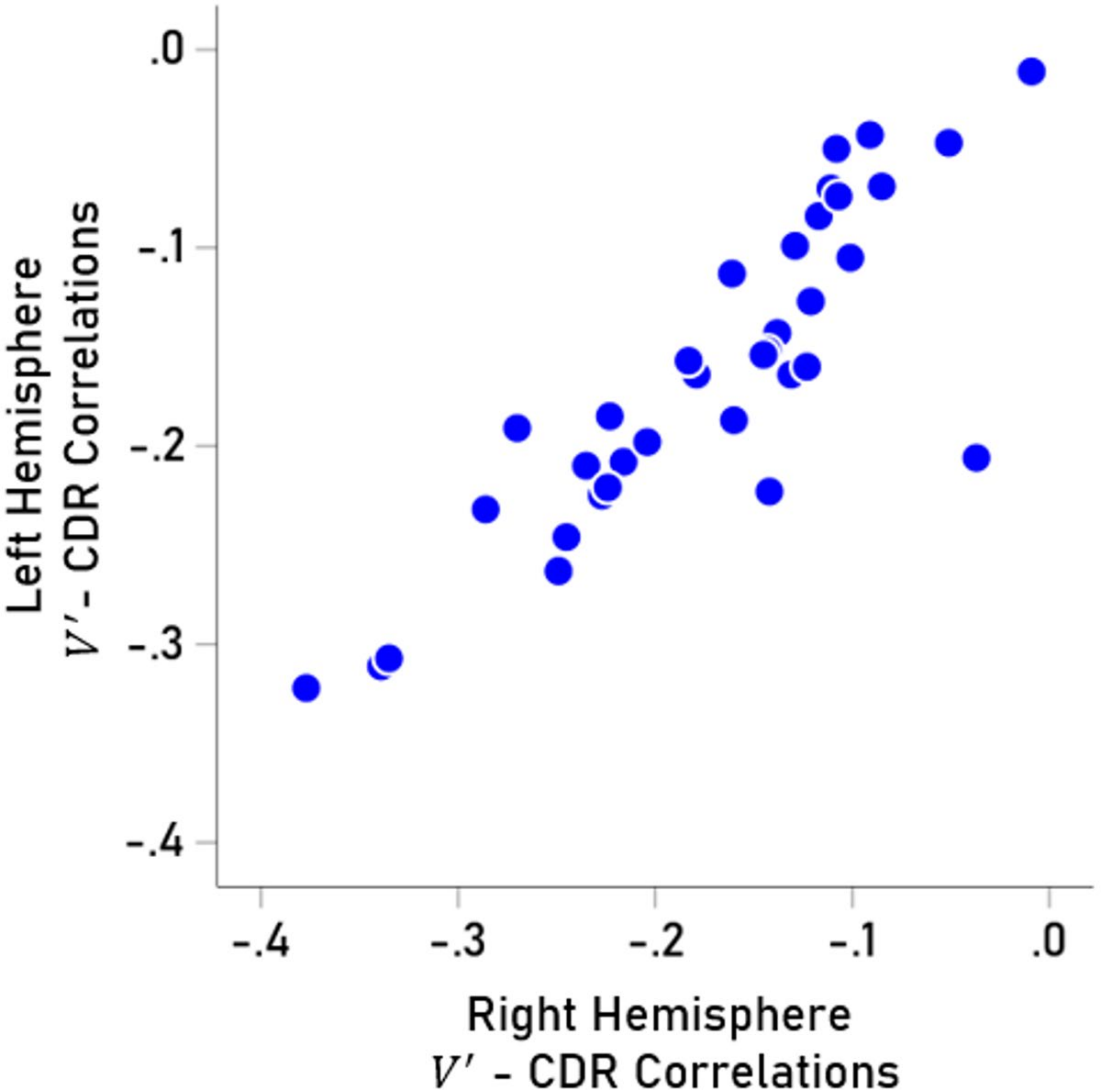
Partial $\:V^{\prime\:}$ - CDR correlations in left vs. right cortical areas (controlling for age and sex). See text for details.

**Fig. 4:**
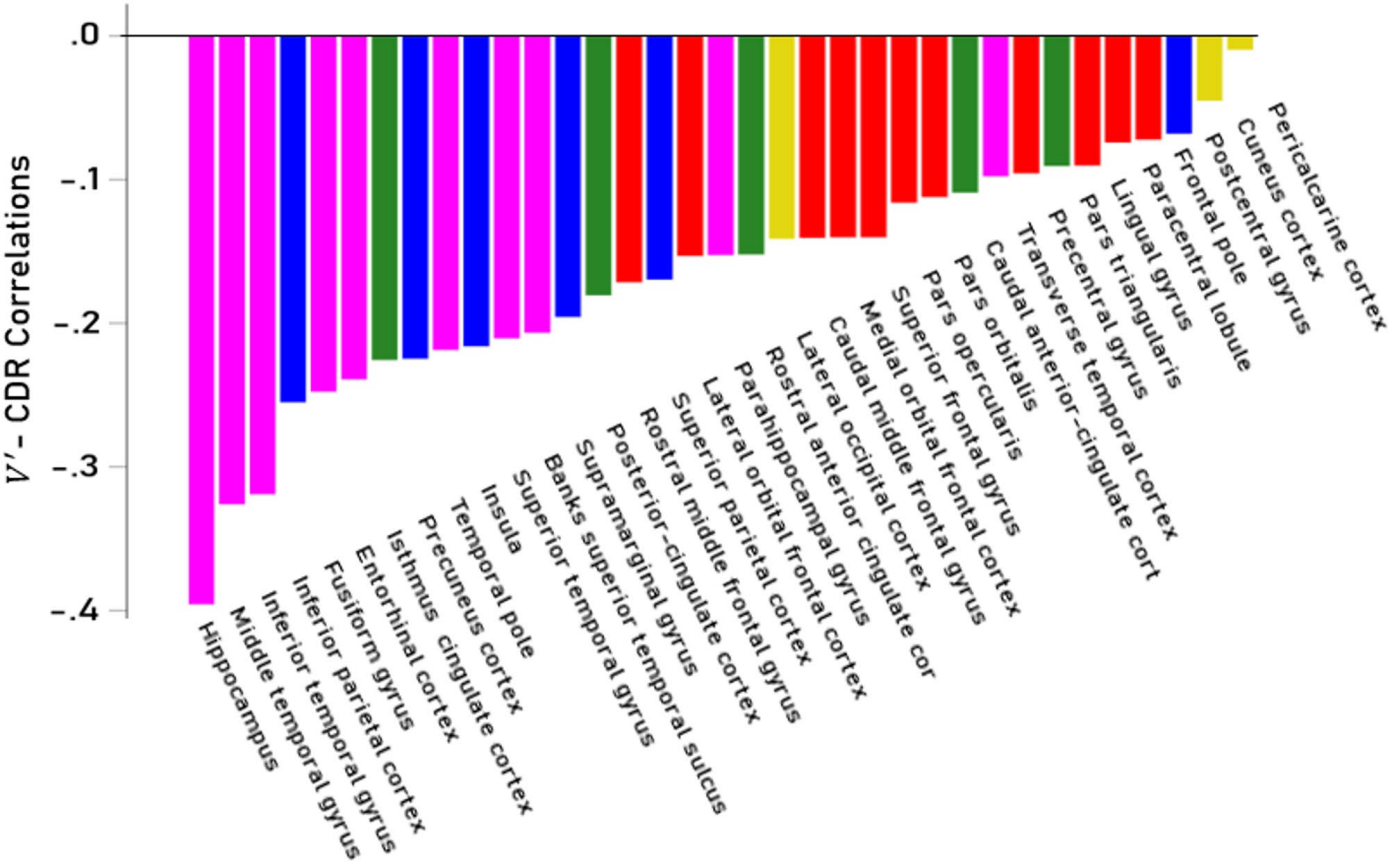
Partial $\:V^{\prime\:}$ - CDR correlations for averaged left and right $\:V^{\prime\:}$ (controlling for age and sex) are shown ranked in a bar graph color-coded to illustrate the cortical lobe of individual areas. Purple, temporal lobe; blue, parietal; green, cingulate; red, frontal; gold, occipital.

**Fig. 5:**
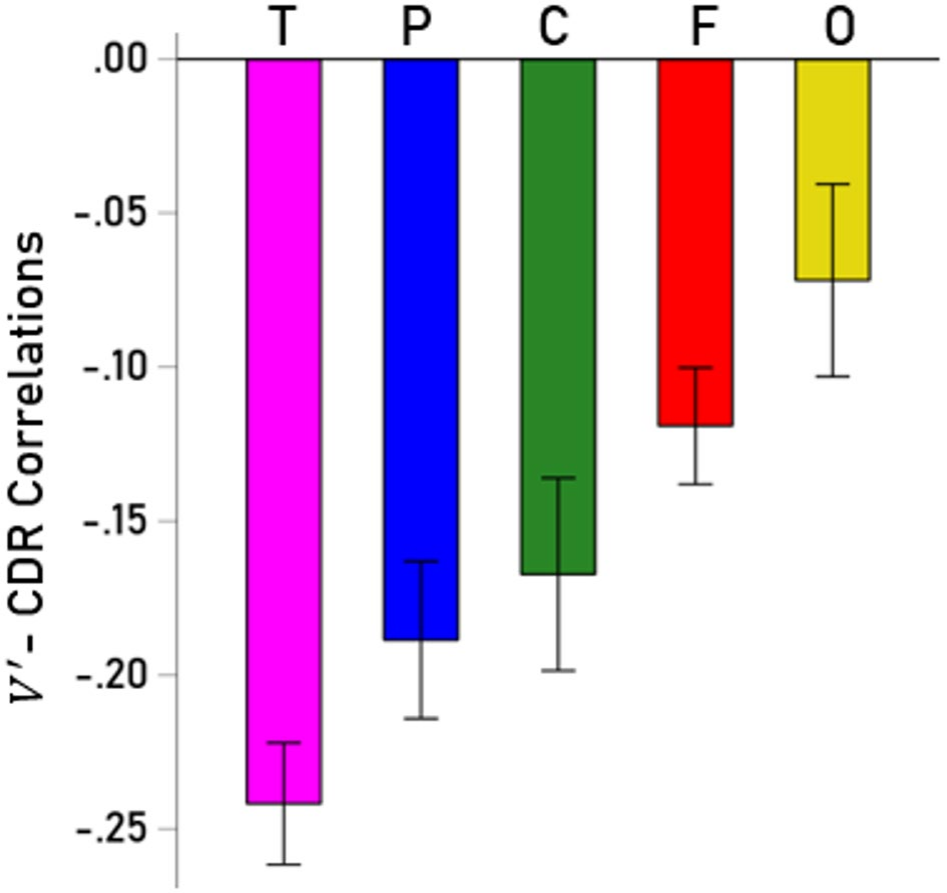
Bar graph of mean (± SEM) partial $\:V^{\prime\:}$ - CDR correlations (controlling for age and sex) for cortical lobes. T, temporal lobe; P, parietal; C, cingulate; F, frontal; O, occipital.

We investigated further the decrease in cortical gray matter by computing partial correlation coefficients between normalized volumes $\:V^{\prime\:}$ and CDR score (controlling for age and sex) for each of the 70 cortical areas analyzed (35 for the left and 35 for the right hemisphere, Table 3). $\:V^{\prime\:}$- CDR partial correlations were highly correlated between left and right hemispheres ($\:r$ = 0.861, *P* < 0.001, *N* = 70; Fig. 3) and did not differ significantly between the two hemispheres (*P* = 0.442, paired-samples t-test), hence were averaged across hemispheres. $\:V^{\prime\:}$ decreased with CDR score most prominently in the hippocampus and least so in the pericalcarine cortex (Fig. 4). The volume decrease was most prominent in the temporal lobe, followed by parietal, cingulate, frontal, and occipital lobes (Fig. 5); volume decreases differed significantly among lobes (*P* < 0.001, F-test, ANOVA).

**Table 3:** Cortical areas analyzed.

	Area	Lobe
1	Banks superior temporal sulcus	Temporal
2	Caudal anterior-cingulate cortex	Cingulate
3	Caudal middle frontal gyrus	Frontal
4	Cuneus cortex	Occipital
5	Entorhinal cortex	Temporal
6	Frontal pole	Frontal
7	Fusiform gyrus	Temporal
8	Hippocampus	Temporal
9	Inferior parietal cortex	Parietal
10	Inferior temporal gyrus	Temporal
11	Insula	Parietal
12	Isthmus cingulate cortex	Cingulate
13	Lateral occipital cortex	Occipital
14	Lateral orbital frontal cortex	Frontal
15	Lingual gyrus	Occipital
16	Medial orbital frontal cortex	Frontal
17	Middle temporal gyrus	Temporal
18	Paracentral lobule	Frontal
19	Parahippocampal gyrus	Temporal
20	Inferior frontal gyrus, pars opercularis	Frontal
21	Inferior frontal gyrus, pars orbitalis	Frontal
22	Inferior frontal gyrus, pars triangularis	Frontal
23	Pericalcarine cortex	Occipital
24	Postcentral gyrus	Parietal
25	Posterior cingulate cortex	Cingulate
26	Precentral gyrus	Frontal
27	Precuneus cortex	Parietal
28	Rostral anterior cingulate cortex	Cingulate
29	Rostral middle frontal gyrus	Frontal
30	Superior frontal gyrus	Frontal
31	Superior parietal cortex	Parietal
32	Superior temporal gyrus	Temporal
33	Supramarginal gyrus	Parietal
34	Temporal pole	Temporal
35	Transverse temporal cortex	Temporal

### Effect of sex

The $\:{V}^{{\prime\:}}$- CDR partial correlations (controlling for age) were significantly correlated ($\:r=$ 0.594, *P* < 0.001, *N* = 70 areas) between men and women. Partial correlations were significantly more negative for men ($\:{r}_{p}\:$= −0.185 ± 0.010, mean ± SEM) than for women ($\:{r}_{p}$= −0.160 ± 0.012) (*P* = 0.018, paired samples t-test).

### Effect of apoE genotype (Tables 4 and 5; Fig. 6)

**Table 4:** Partial correlation coefficients of $\:V^{\prime\:}$ - CDR for areas of the left hemisphere (controlling for age and sex).

	Area	apoE[ε2ε2,ε2ε3]	apoE[ε3ε3]	apoE[ε4ε4,ε3ε4]
1	Banks superior temporal sulcus	−0.094	−0.234	−0.280
2	Caudal anterior-cingulate cortex	−0.144	−0.051	0.003
3	Caudal middle frontal gyrus	−0.187	−0.196	−0.092
4	Cuneus cortex	−0.095	−0.107	−0.033
5	Entorhinal cortex	−0.412	−0.179	−0.272
6	Frontal pole	−0.035	−0.112	−0.141
7	Fusiform gyrus	−0.144	−0.216	−0.355
8	Hippocampus	−0.473	−0.309	−0.501
9	Inferior parietal cortex	−0.255	−0.278	−0.338
10	Inferior temporal gyrus	−0.331	−0.350	−0.344
11	Insula	−0.029	−0.174	−0.359
12	Isthmus cingulate cortex	−0.205	−0.210	−0.343
13	Lateral occipital cortex	−0.109	−0.119	−0.197
14	Lateral orbital frontal cortex	−0.098	−0.152	−0.145
15	Lingual gyrus	−0.106	−0.055	−0.193
16	Medial orbital frontal cortex	−0.244	−0.105	−0.090
17	Middle temporal gyrus	−0.153	−0.326	−0.421
18	Paracentral lobule	−0.030	−0.138	−0.064
19	Parahippocampal gyrus	−0.247	−0.083	−0.178
20	Inferior frontal gyrus, pars opercularis	−0.130	−0.154	−0.089
21	Inferior frontal gyrus, pars orbitalis	−0.086	−0.165	−0.106
22	Inferior frontal gyrus, pars triangularis	−0.057	−0.096	−0.150
23	Pericalcarine cortex	−0.112	0.022	−0.050
24	Postcentral gyrus	−0.055	−0.039	−0.151
25	Posterior cingulate cortex	−0.052	−0.142	−0.159
26	Precentral gyrus	−0.202	−0.118	−0.073
27	Precuneus cortex	−0.075	−0.210	−0.272
28	Rostral anterior cingulate cortex	−0.229	−0.255	−0.112
29	Rostral middle frontal gyrus	−0.200	−0.108	−0.229
30	Superior frontal gyrus	−0.157	−0.107	−0.248
31	Superior parietal cortex	−0.063	−0.137	−0.244
32	Superior temporal gyrus	−0.073	−0.186	−0.257
33	Supramarginal gyrus	0.007	−0.206	−0.265
34	Temporal pole	−0.249	−0.181	−0.221
35	Transverse temporal cortex	−0.098	−0.077	−0.121

**Table 5:** Partial correlation coefficients of $\:V^{\prime\:}$ - CDR for areas of the right hemisphere (controlling for age and sex).

	Area	apoE[ε2ε2,ε2ε3]	apoE[ε3ε3]	apoE[ε4ε4,ε3ε4]
1	Banks superior temporal sulcus	−0.180	−0.213	−0.227
2	Caudal anterior-cingulate cortex	−0.267	−0.133	−0.288
3	Caudal middle frontal gyrus	−0.237	−0.207	−0.116
4	Cuneus cortex	−0.121	−0.115	−0.005
5	Entorhinal cortex	−0.425	−0.158	−0.308
6	Frontal pole	0.062	0.079	−0.200
7	Fusiform gyrus	−0.160	−0.158	−0.371
8	Hippocampus	−0.322	−0.259	−0.484
9	Inferior parietal cortex	−0.118	−0.196	−0.306
10	Inferior temporal gyrus	−0.331	−0.258	−0.350
11	Insula	−0.248	−0.194	−0.254
12	Isthmus cingulate cortex	−0.120	−0.154	−0.238
13	Lateral occipital cortex	−0.079	−0.158	−0.179
14	Lateral orbital frontal cortex	−0.174	−0.115	−0.159
15	Lingual gyrus	−0.056	−0.036	−0.126
16	Medial orbital frontal cortex	−0.326	−0.039	−0.234
17	Middle temporal gyrus	−0.193	−0.271	−0.372
18	Paracentral lobule	−0.027	−0.034	−0.131
19	Parahippocampal gyrus	−0.304	−0.089	−0.144
20	Inferior frontal gyrus, pars opercularis	−0.066	−0.152	−0.083
21	Inferior frontal gyrus, pars orbitalis	−0.138	−0.092	−0.105
22	Inferior frontal gyrus, pars triangularis	−0.078	−0.068	−0.118
23	Pericalcarine cortex	−0.050	−0.061	−0.010
24	Postcentral gyrus	−0.019	−0.022	−0.043
25	Posterior cingulate cortex	−0.130	−0.169	−0.320
26	Precentral gyrus	−0.140	−0.074	−0.076
27	Precuneus cortex	0.045	−0.215	−0.239
28	Rostral anterior cingulate cortex	−0.211	−0.198	−0.146
29	Rostral middle frontal gyrus	−0.251	−0.150	−0.228
30	Superior frontal gyrus	−0.091	−0.058	−0.210
31	Superior parietal cortex	0.076	−0.215	−0.143
32	Superior temporal gyrus	−0.178	−0.150	−0.254
33	Supramarginal gyrus	−0.087	−0.186	−0.174
34	Temporal pole	−0.184	−0.152	−0.260
35	Transverse temporal cortex	−0.155	−0.049	−0.126

**Fig. 6:**
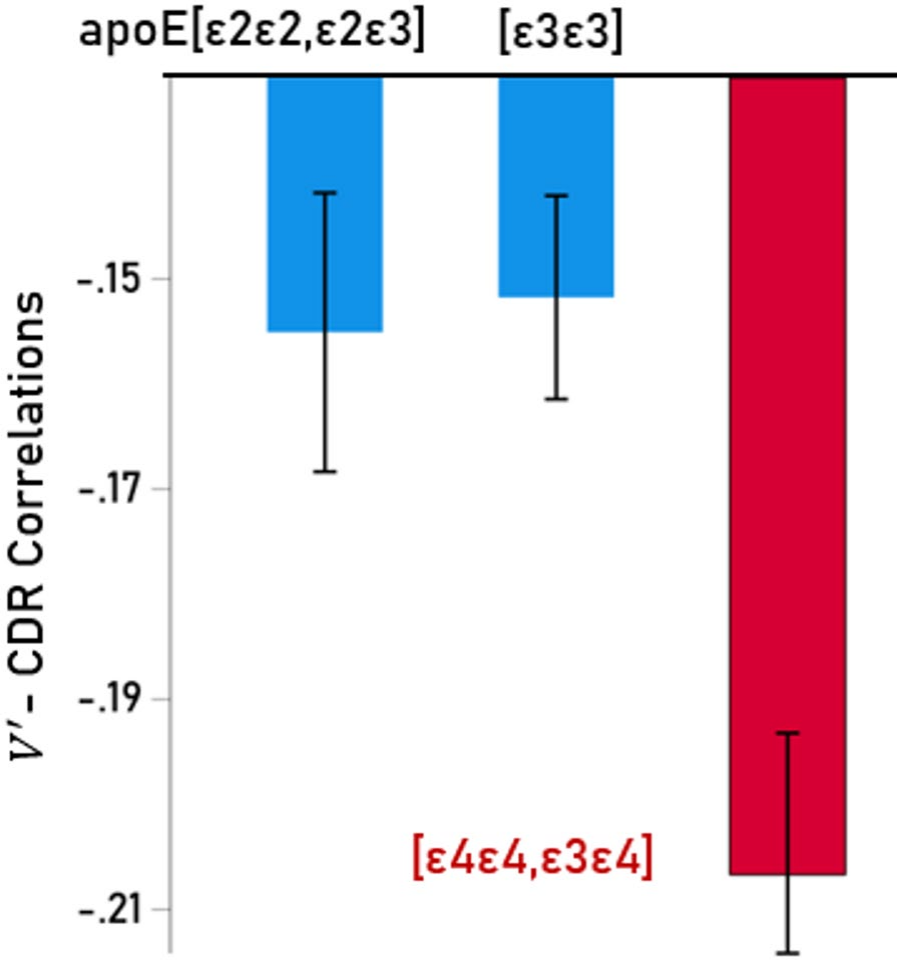
Mean (± SEM) partial $\:V^{\prime\:}$ - CDR correlations for the three apoE groups analyzed, as indicated.

The effect of apoE group on $\:{V}^{{\prime\:}}$- CDR partial correlations $\:{r}_{p}$was assessed by performing a repeated measures ANOVA where the partial correlations were the repeated measures factor for each cortical area (*N* = 70). We found the following. (a) The apoE effect was statistically significant (*P* < 0.001, Greenhouse-Geisser test). (b) The mean $\:{V}^{{\prime\:}}$- CDR partial correlation ($\:{r}_{p}$ = −0.202) for the apoE[ε4ε4,ε4ε3] ($\:{r}_{p}$ = −0.202) group was significantly more negative than that of the apoE[ε3ε3] ($\:{r}_{p}$ = −0.147, *P* < 0.001, Bonferroni adjusted) and that of the apoE[ε2ε2,ε2ε3] ($\:{r}_{p}$ = −0.150, *P* = 0.002, Bonferroni adjusted). (c) The mean $\:{V}^{{\prime\:}}$- CDR partial correlations did not differ significantly between the apoE[ε3ε3] and the apoE[ε2ε2,ε2ε3] groups (*P* = 1.0, Bonferroni adjusted).

### Subcortex

#### CDR and subcortical nuclei

**Fig. 7:**
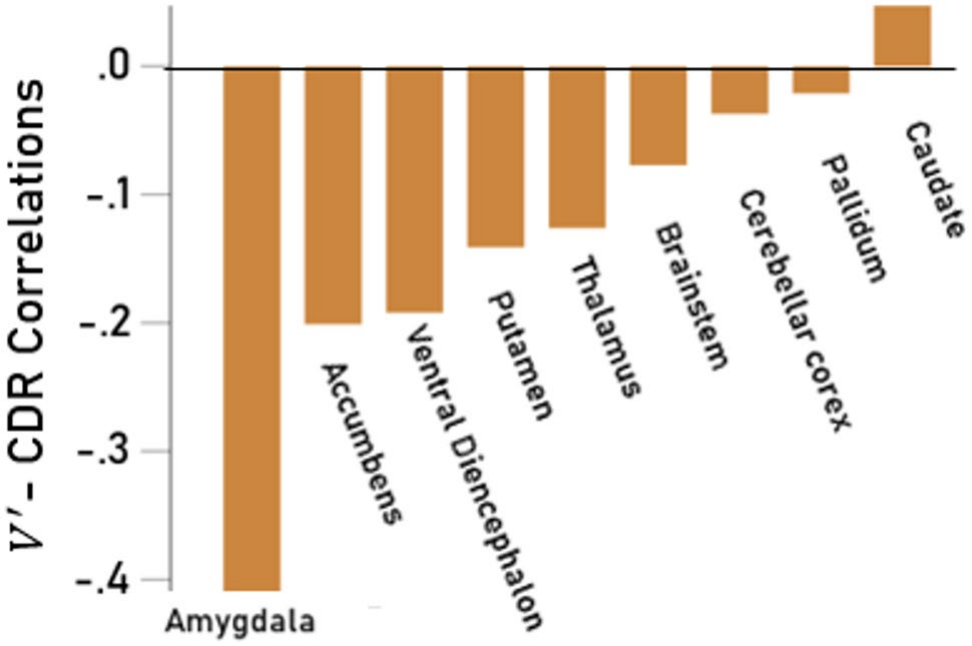
Subcortical volumes: Partial $\:V^{\prime\:}$ - CDR correlations for averaged left and right $\:V^{\prime\:}$ (controlling for age and sex) for subcortical nulcei. See text for details.

We computed partial correlation coefficients between normalized subcortical volumes $\:V^{\prime\:}$ and CDR score (controlling for age and sex) for each of the 17 subcortical areas analyzed (8 for the left hemisphere, 8 for the right hemisphere, and the brainstem, Table 6). Volume-CDR partial correlations were highly correlated between left and right hemispheres ($\:r$ = 0.971, *P* < 0.001, *N* = 8) and did not differ significantly between the two hemispheres (*P* = 0.153, paired-samples t-test), hence were averaged across hemispheres. Volume decreased with CDR score most prominently in the amygdala and least in the pallidum (Fig. 7); paradoxically, caudate volume increased (not significantly) with CDR score.

**Table 6:** Subcortical nuclei analyzed.

	Nucleus
1	Accumbens
2	Amygdala
3	Brainstem
4	Caudate
5	Cerebellar cortex
6	Pallidum
7	Putamen
8	Thalamus
9	Ventral diencephalon

### Effect of sex

The $\:{V}^{{\prime\:}}$- CDR partial correlations (controlling for age) were significantly correlated between men and women ($\:r=$ 0.761, *P* < 0.001, *N* = 17 subcortical nuclei) and did not differ significantly between the two groups (*P* = 0.099, paired-samples t-test).

### Effect of ApoE genotype

**Fig. 8:**
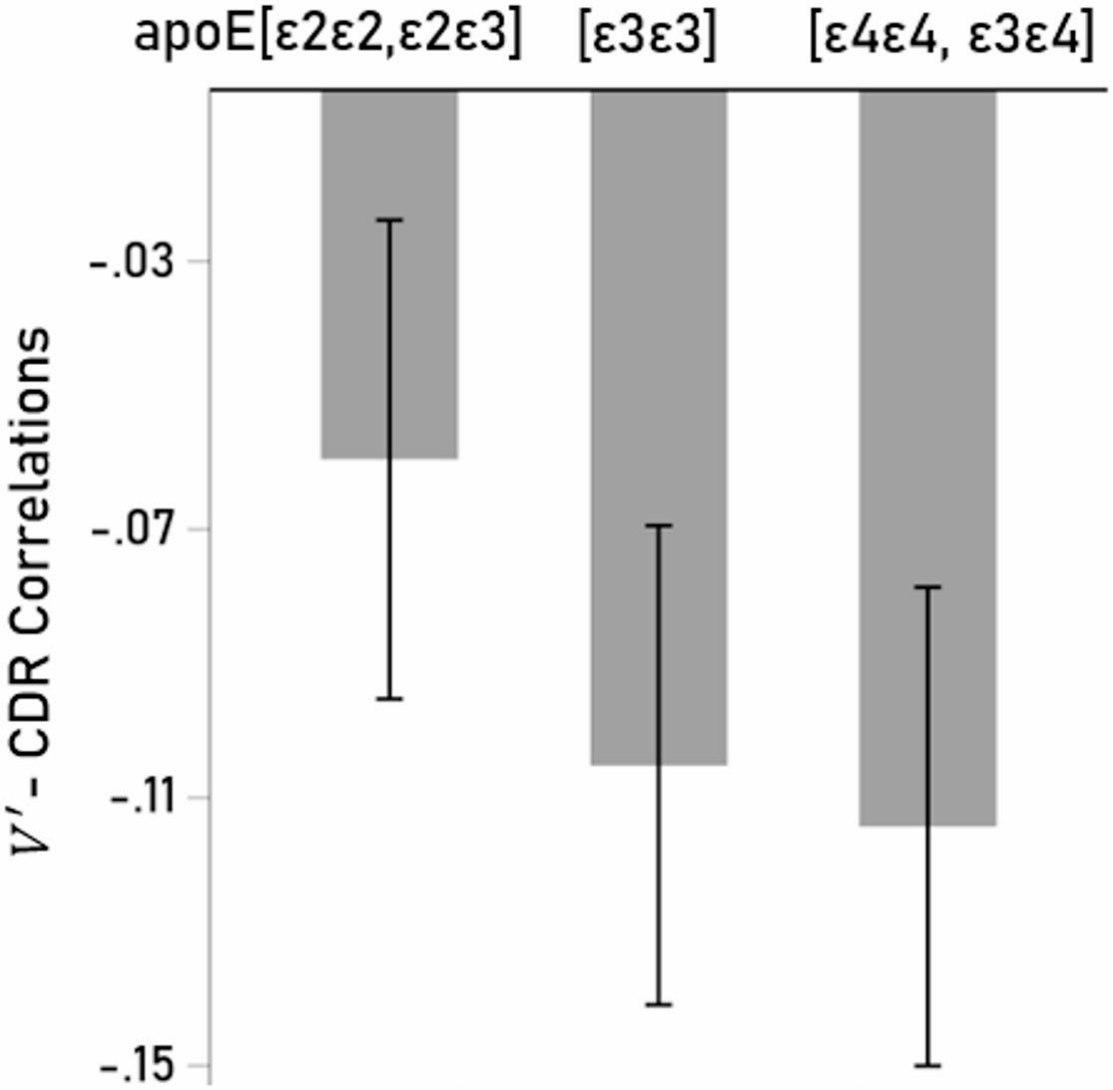
Subcortical volumes: Mean (± SEM) partial $\:V^{\prime\:}$ - CDR correlations for the three apoE groups analyzed, as indicated. See text for details.

The effect of apoE group on subcortical $\:{V}^{{\prime\:}}$- CDR partial correlations $\:{r}_{p}$was assessed by performing a repeated measures ANOVA where the partial correlations were the repeated measures factor for each cortical area. Although correlations were least negative in the control group (CDR 0; Fig. 8), none of the pairwise comparisons in the ANOVA attained statistical significance (*P* ≥ 0.243).

#### Multivariate analyses of brain volumes and CDR: linear discriminant analysis (LDA)

In these analyses we used all averaged 44 brain volumes (35 cortical and 9 subcortical), adjusted for age and sex, to evaluate whether CDR staging can be predicted by a linear combination of the 44 brain volumes (adjusted for sex and age). We found the following.

### Control vs. dementia groups

**Fig. 9:**
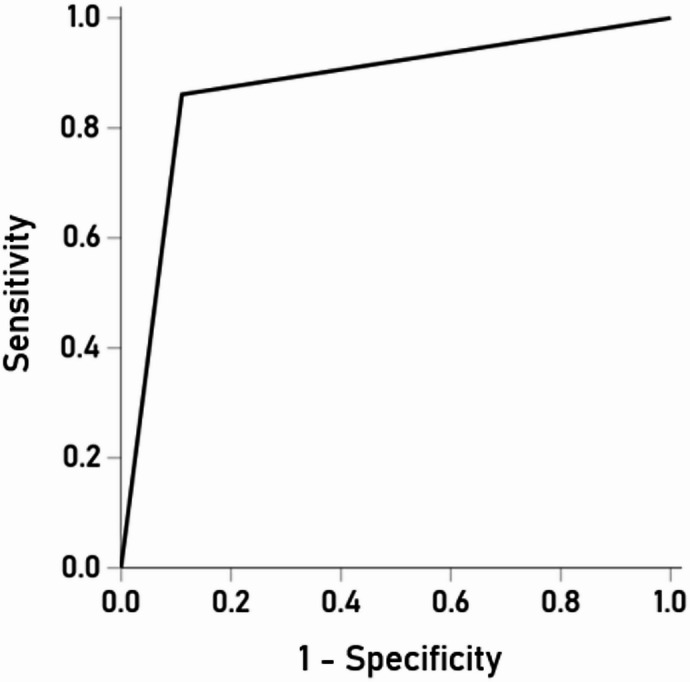
ROC curve of Control vs. Dem LDA.

The Control (CDR 0) and Dementia (CDR 1) groups are defined unequivocally, in contrast to the “undetermined” group (CDR 0.5) which is a heterogeneous, transitional group. Therefore, we first performed a LDA between the Control and Dementia groups, the results of which are shown in Table 7. It can be seen that the discrimination was excellent, with an overall correct classification rate of 88.7% (88.9% sensitivity and 86.1% specificity). These results were statistically highly significant (Wilk’s Λ = 0.661, *P* < 0.001). The ROC curve is shown in Fig. 9; area = 0.875, SE = 0.035, *P* < 0.001).

**Table 7:** LDA classification results for control (CDR 0) vs. Dementia (CDR 1).

		Predicted		Total
		Control	Dementia	
Actual	Control	313	39	352
	Dementia	5	31	36
		Percent (%)		
Actual	Control	**88.9**	11.1	100
	Dementia	13.9	**86.1**	100
Overall correct classification: **88.7%**				

**Fig. 10:**
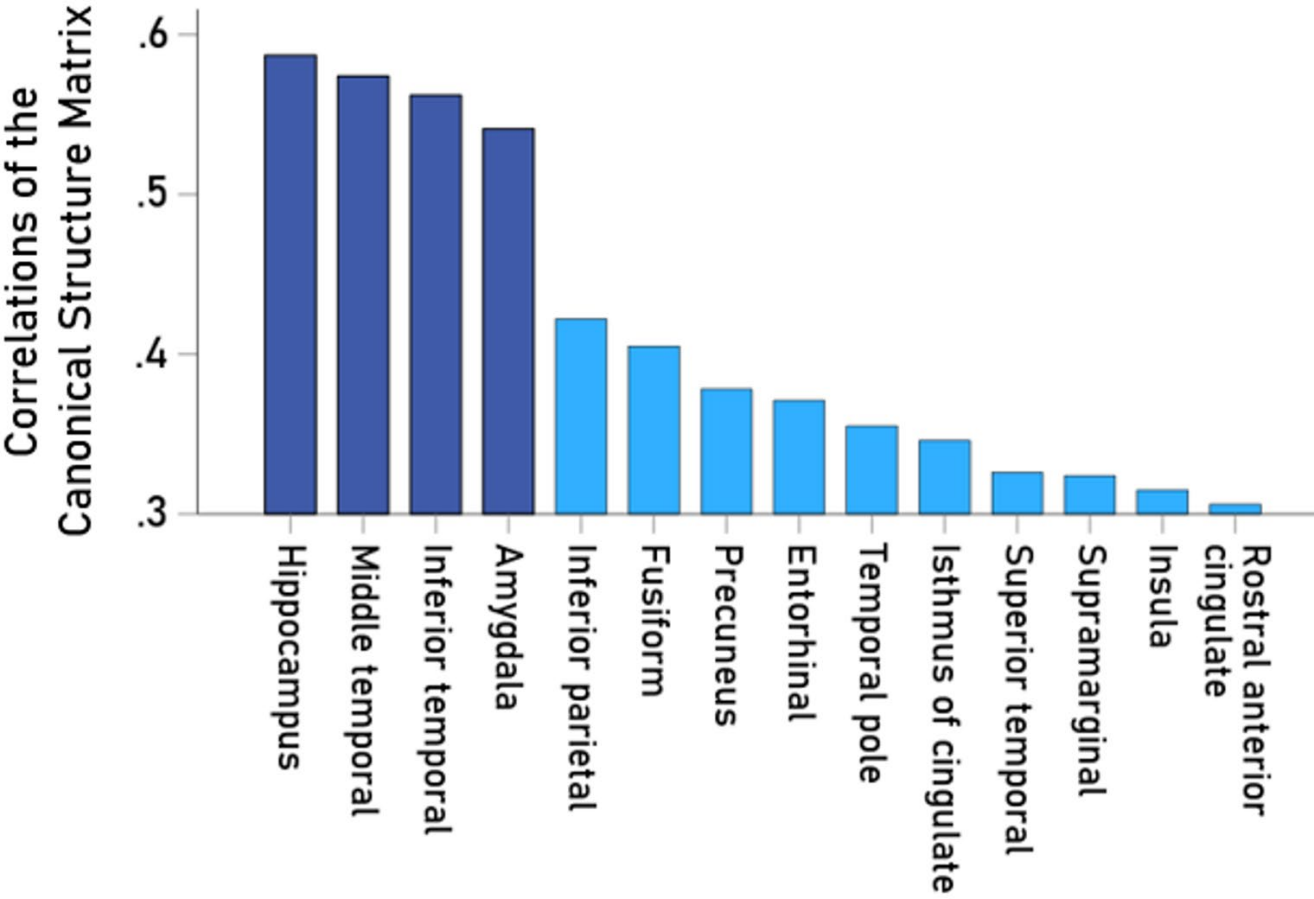
Bar graph of LDA structure function of Control vs. Dementia, indicating the relative contribution of each brain area to the discrimination of these groups. In general, variables with values > 0.3 are considered important contributors to the discrimination.

The bar graph in Fig. 10 shows the correlations in the LDA structure matrix where the magnitude of the values (bar length) indicates the relative contribution of each area to the group discrimination; in general values > 0.3 are considered important contributors to the discrimination. It can be seen that areas with high values were also strongly associated with CDR (Figs. 4 and 7).

#### *Two subgroups in the “undetermined” (CDR 0.5) group*

**Fig. 11:**
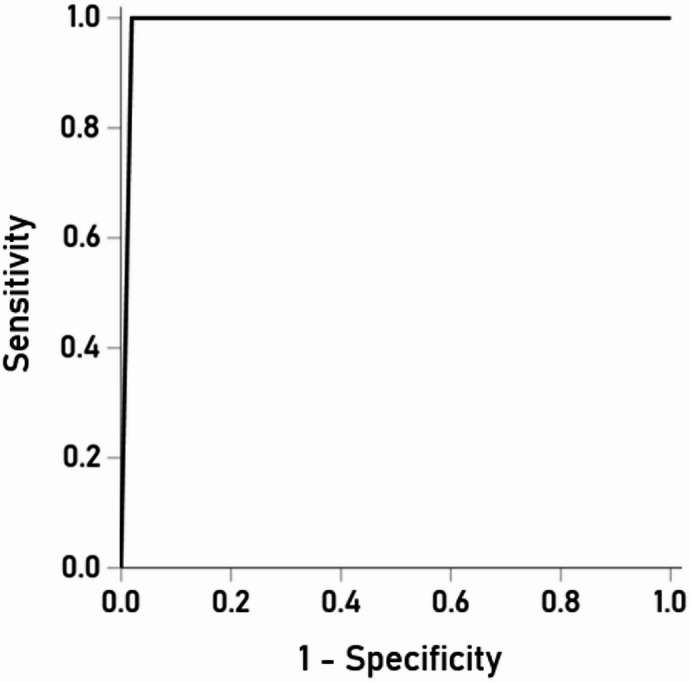
ROC curve of validation of CDR 0.5 cases based on discriminant function derived from Control/Dementia LDA.

The results above provide a solid framework to test the hypothesis that, *based on brain volumes*, the “undetermined” group (CDR 0.5) may actually consist of 2 distinct cluster, Cluster A (closer to the Control group) and Cluster B (closer to the Dementia group). We tested this hypothesis by using the classification functions yielded by the Control vs. Dementia LDA above to classify participants in the “undetermined” group. This analysis yielded 2 clusters; Cluster A comprised 51 (70.8%) brains that were assigned to the Control group, whereas Cluster B comprised 21 (29.2%) brains that were assigned to the Dementia group. A LDA using them as the dependent, grouping variable and the 44 brain volumes as predictors gave excellent classification results shown in Table 8. The overall correct classification rate was 98.6%, with 98.0% specificity and 100% sensitivity. The ROC curve is shown in Fig. 11; area = 0.990, SE = 0.011, *P* < 0.001). Remarkably, the probabilities of classification of a brain to either Cluster A or B were very high, with 66/72 (91.7%) been 1.0, and the rest 6 > 0.97 (Table 9). This high reliability of dichotomous classification was further documented by calculating the Squared Mahalanobis Distance (SMD) of a case from the center of each Cluster. The mean SMD (± SEM) from the center of the Cluster to which a case was classified was 0.874 (± 0.126, *N* = 72), as compared to the SMD from the center of the other Cluster 31.12 (± 1.213, *N* = 72); this 36x difference was statistically highly significant (*P* = 8.8 × 10^−37^, t_[71]_ = 27.87, paired t-test, *N* = 72). Altogether, these results document the high reliability of dichotomous classification of brains in the “undetermined” CDR 0.5 group to 2 Clusters (Control-like and Dementia-like).

**Table 8:** Validation of classification of CDR 0.5 cases based on discriminant functions from control vs. Dementia LDA classification. See text for details.

		Predicted CDR 0.5 LDA		Total
		Control	Dementia	
Predicted by Control-Dementia LDA	Control	50	1	51
	Dementia	0	21	21
		Percent (%)		
Predicted by Control-Dementia LDA	Control	**98.0**	2.0	100
	Dementia	0.0	**100.0**	100
Overall correct classification: **98.6%**				

**Table 9:** Frequency distribution of probabilities of classification of a brain in the “undetermined” (CDR 0.5) to cluster A (Control-like) or cluster B (Dementia-like). See text for details.

Probability	Frequency	Percent
0.972	1	1.4
0.981	1	1.4
0.995	1	1.4
0.997	2	2.8
0.999	1	1.4
1.000	66	91.7
Total	72	100

## Discussion

### General

Here we evaluated cortical and subcortical gray matter volumes in cognitively unimpaired individuals (CDR 0) and those with undetermined (CDR 0.5) and early dementia (CDR 1) in a cross sectional sample. We found a systematic decrease in global cortical gray matter volumes with increasing CDR score that was most prominent in the temporal lobe, and was modulated by sex and apoE genotype. Subcortical gray matter atrophy volumes also decreased with increasing CDR score for all nuclei except for the caudate; the decrease was most prominent in the amygdala. The highly significant cortical and subcortical gray matter volume reduction with increasing CDR scores supports the sensitivity of the CDR, a semi-structured interview evaluating cognitive and functional decline, in capturing impairments associated with brain volume loss, the most proximal indicator of dementia progression^37^, even at the earliest stages.

### Methodological considerations: partial correlation

The statistical measure used in this study is the partial correlation, which enabled us to assess/quantify the effect of CDR on brain volumes, independently of (i.e. while removing, controlling for) effects of other factors (e.g. age and sex) that could also affect the volumes.

The partial correlation is indeed the statistical tool by which to evaluate the effect of an independent variable on a dependent variable, in the presence of other independent variables that could also affect the dependent variable. Consider the case of coronary artery disease, as the dependent variable. We know that several independent variables can contribute to it, including genetics, diet, exercise, smoking etc. How do we assess the effect of lipids in the presence of all the other variables? The general solution to this problem was provided by Udny Yule, professor of statistics at Cambridge University, UK, who invented in 1897 the “partial regression analysis”^38^ in which the effect of a number of independent variables on the dependent variable is removed from the data by holding one or more independent variables constant during the analysis, thus estimating the effect of a specific independent variable on the dependent variable *controlling for the effect of the other variables.* A formal treatment of partial correlation and its distribution was provided by Fisher in 1924^39^. Lucid descriptions of the concept and applications of the partial correlation have been published^40,41^, and its calculation can be found in standard textbooks of statistics^42–44^ and computer packages of statistical analyses [SPSS, Matlab, R, etc.]. Partial correlation analysis has had a tremendous impact on epidemiology and medicine, where there is hardly an outcome of a disease (acute or chronic, infectious or not, pediatric or geriatric, etc.) that can be attributed to just one and only one factor. Diseases are typically multifactorial (i.e. influenced by several factors) and it is the tool of partial correlation that teases out the contribution of each factor to the appearance, severity, trajectory, and ultimate effect of the disease on health and survival. The same multifactorial considerations apply to the design and evaluation of treatments and clinical trials of various therapies. In summary, partial correlation is a powerful tool for assessing the effect of an independent variable on a depended variable, while controlling for possible effects of other independent variables, as we applied in this study to evaluate the effect of CDR on brain volumes, while controlling for age and sex.

### Change of brain volumes along the CDR scale

Compared to cognitively unimpaired individuals, significantly reduced cortical gray matter volume was documented in individuals with a CDR score of 0.5, highlighting gross structural changes amongst those with undetermined or questionable dementia that increased in scope and severity among those with mild dementia (CDR 1). In contrast, a previous study did not find global gray matter volume reductions associated with CDR 0.5 but did report volume reduction in several regions including left olfactory gyrus, left amygdala, left inferior temporal gyrus, right superior temporal gyrus, and bilateral putamen^32^. With regard to specific areas, we also found evidence of widespread volume reduction with increasing CDR; specifically, volumes of the middle and inferior temporal gyri were most strongly associated with increased CDR followed by inferior parietal cortex, fusiform gyrus, and entorhinal cortex. This emphasis on temporal cortical lobe volume reductions (4 out of the top 5 areas) with increasing early CDR scores is consistent with a broad literature indicating the medial temporal lobe as typically the first area to show evidence of dementia-associated atrophy^7^. Furthermore, we found that the association between cortical volume reduction and CDR scores was strongest in the temporal lobe followed by parietal, cingulate, frontal, and occipital lobe, consistent with the typical progression of atrophy with increasing dementia severity^7,10,12,45–48^. It is noteworthy that all 34 cortical brain areas were characterized by cortical volume reductions with increased CDR scores in this sample that represented only cognitively unimpaired, undetermined dementia, and mild dementia, highlighting the pervasiveness of cortical gray matter volume loss even at these early stages.

Similarly, subcortical volume decreased with increased CDR scores for all nuclei with the exception of the caudate. Several prior studies have documented subcortical nuclei atrophy with dementia progression that is evident even in the preclinical stages^7,14,16^. In particular, volume loss in the amygdala has been widely reported^7,15^, is evident even at very mild stages of dementia (CDR 0.5), and amygdala volume has been shown to discriminate between dementia and controls^48^. Volume reduction of other subcortical areas such as the thalamus, putamen, and nucleus accumbens have also been documented^7,14,49^, particularly at more advanced stages of the disease^14^. In contrast, even moderate stages of AD are not associated with volume changes in the pallidum^7,14^, consistent with the modest association documented here. Previous findings with regard to the caudate have been inconsistent. While some studies have reported reduced caudate volume in MCI and AD compared to controls^16,50^, others have reported no differences in between cases and controls^14^, suggesting the caudate may be relatively preserved in the early stages of the disease relative to other areas^49^. Consistent with our findings is evidence of increased caudate volume, particularly during the early stages of dementia^51,52^, which has been attributed to hypertrophy, a compensatory response, and/or inflammation resulting from AD pathology early in the disease process^51^. Recent evidence suggest that the ratio of cortical volume to caudate volume may be a particularly useful indicator of cognitive decline associated with dementia^53^.

### Effect of sex and ApoE genotype

Cortical volume reduction rates were very similar in men and women, and did not differ significantly between sexes. By contrast, the apoE[ε4ε4,ε3ε4] group was characterized by much stronger associations between cortical volume reduction and CDR staging than the apoE[ε2ε3] or the apoE[ε3ε3] groups which did not differ from each other. The excess reduction in cortical volume with increased CDR scores in the apoE[ε4ε4,ε3ε4] group is in line with robust evidence of apoE4 as a key genetic risk factor for dementia^54–56^, accounting for 7% of overall dementia risk^57^. In contrast, there were no differences in subcortical volume associations with CDR for the apoE groups. As reviewed elsewhere^58^, some but not all previous studies have documented atrophy associated with apoE4 in AD patients, with most evidence pointing toward apoE4 effects primarily involving the medial temporal lobe. Some have found apoE4 effects on volume are limited to specific brain areas (vs. global atrophy)^59^, those with AD (vs. MCI or controls)^60^, specific AD subtypes^61^, and age of onset^62^. Thus, apoE4 effects on subcortical nuclei may not be evident in the earliest stages of the dementia continuum investigated here.

### The dementia “undetermined” group (CDR 0.5)

Brain atrophy is a hallmark of dementia. Here, brain volume accurately discriminated between individuals characterized as controls (CDR 0) or dementia (CDR 1), similar to findings from other studies documenting highly accurate classification of AD from controls based on MRI-derived structural measures^63^. Applying those classification functions to the Undetermined (CDR 0.5) participants, however, yielded two distinct clusters, with 51/72 (70.8%) classified as Controls and 21/72 (29.2%) as Dementia. Remarkably, the group classification probabilities were very high (Table 9), further solidifying the dichotomous composition of this group.

Substantial research has documented that MCI, equivalent to CDR 0.5, is a notoriously heterogeneous group in which some will go on to develop dementia and others will not^6,64^. The present findings indicate that structural MRI, particularly when coupled with the CDR or other similar measures, may be useful in identifying individuals with MCI who are potentially at risk for developing dementia and who may benefit from monitoring and potentially early intervention.

### Limitations

A major limitation of this study is the unequal sample sizes. We believe that the most important aspect is that the N in the control group (CDR 0) is sufficiently large (*N* = 352) to allow detection/evaluation of differences in the “disease” groups. (The opposite balance would have been problematic.) In addition, since these results are based on a cross-sectional sample, they should be regarded as a hypothesis-generating observations that remain to be validated in longitudinal studies.

## Data Availability

Data are publicly available from the websites mentioned in the Materials and Methods section. OASIS-3 (Open Access Series of Imaging Studies): Longitudinal Multimodal Neuroimaging, www.oasis-brains.org (https://sites.wustl.edu/oasisbrains/).
